# Inhibition of PGE_2_/EP4 receptor signaling enhances oxaliplatin efficacy in resistant colon cancer cells through modulation of oxidative stress

**DOI:** 10.1038/s41598-019-40848-4

**Published:** 2019-03-20

**Authors:** Huakang Huang, Oladimeji Aladelokun, Takayasu Ideta, Charles Giardina, Lee M. Ellis, Daniel W. Rosenberg

**Affiliations:** 1 Center for Molecular Oncology, University of Connecticut Health, 263 Farmington Ave, Farmington, CT USA; 2 Department of Cell and Molecular Biology, University of Connecticut, Storrs, CT USA; 3 Department of Surgical Oncology, University of Texas MD Anderson Cancer Center, Holcombe Boulevard, Houston, Texas USA

## Abstract

The platinum-based chemotherapeutic agent, oxaliplatin, is used to treat advanced colorectal cancer (CRC). Unfortunately, nearly all patients acquire resistance to oxaliplatin after long-term use, limiting its therapeutic efficacy. Since COX-2 and PGE_2_ signaling can impact colon cancer cell proliferation and survival, we examined how this pathway was affected in an oxaliplatin resistant colon cancer cell line. PGE_2_ levels were significantly elevated in oxaliplatin-resistant HT29 cells (OXR) compared to naïve parental HT29 cells (PAR). This increase was associated with elevated COX-2 (17.9-fold; P = 0.008) and reduced 15-hydroxyprostaglandin dehydrogenase (2.9-fold; P < 0.0001) expression. RNAi knockdown of microsomal prostaglandin E synthase-1, the rate-limiting enzyme in PGE_2_ synthesis, sensitized OXR cells to oxaliplatin. Downstream effects of PGE_2_ in OXR cells were also examined. Selective inhibition of the EP4 PGE_2_ receptor by the small molecule inhibitor, L-161,982 enhanced oxaliplatin-induced apoptosis in OXR cells. L-161,982 also reduced expression of the colonic stem cell markers, CD133 and CD44, and inhibited tumor sphere formation. The accumulation of intracellular reactive oxygen species (ROS), a key component of oxaliplatin cytotoxicity, was significantly increased by EP4 inhibition (2.4 -fold; P < 0.0001). Overall, our findings uncover an important role for the COX-2/PGE_2_/EP4 signaling axis in oxaliplatin resistance *via* regulation of oxidative stress.

## Introduction

Colorectal cancer (CRC) is the third most commonly diagnosed cancer and the third leading cause of cancer-related deaths in the United States^1^. Advances in cancer prevention efforts, including the widespread application of screening colonoscopy along with the identification and removal of precancerous lesions, have led to a significant overall reduction in CRC incidence^2–5^. However, available treatment options for advanced CRC often fail, generally due to the acquisition of chemoresistance^6^. Oxaliplatin, a third-generation platinum derivative, exhibits strong activity against CRC and has been widely used as a first-line chemotherapeutic agent together with 5-fluorouracil and leucovorin (FOLFOX) for the treatment of metastatic CRC^7,8^. Oxaliplatin covalently binds to DNA to form cross-links, leading to cell cycle arrest, and apoptosis^9,10^. Although the clinical response rate to oxaliplatin is approximately 24%, acquired resistance develops in nearly all patients after long-term treatment with either oxaliplatin alone, or with FOLFOX, ultimately limiting its therapeutic efficacy^6,11.^ Establishing a clearer understanding of mechanisms that contribute to oxaliplatin resistance is imperative for developing more effective therapeutic strategies that may overcome drug resistance and enhance oxaliplatin efficacy.

Prostaglandin E_2_ (PGE_2_) is a bioactive lipid metabolite that elicits a wide range of biological effects associated with inflammation and cancer^12–15^. A number of clinical and pre-clinical studies have shown that the long-term use of non-steroidal anti-inflammatory drugs (NSAIDs) is an effective approach for CRC prevention, largely due to the blockade of PGE_2_ synthesis *via* inhibition of the cyclooxygenases, COX-1 and COX-2^16–18^. In fact, several studies have shown that targeting PGE_2_ synthesis enhances the response to conventional and targeted chemotherapies^19–21^, and drug combinations with COX inhibitors have been shown to overcome chemo-resistance found in bladder and metastatic breast cancers^22–24^. Other studies have also shown a synergistic response to COX-2 inhibitors when used in combination with oxaliplatin or 5-FU^19,20,25^. In this study, we examined how PGE_2_ production and downstream signaling is affected in an oxaliplatin-resistant colon cancer cell line. Our findings uncover an important role for the COX-2/PGE_2_/EP4 signaling axis in chemoresistance, in part through regulating the cellular redox status. These studies provide the basis for further investigation into targeting EP4 as an adjuvant therapy for increasing oxaliplatin efficacy in CRC patients.

## Materials and Methods

### Cell lines and culture conditions

The human CRC cell lines HT29, RKO, SW480, Caco-2 and HCT116 were obtained from the American Type Culture Collection. The oxaliplatin-resistant cell lines HT29 OXR and RKO OXR were generated as previously described^26^. Briefly, chemo-naïve HT29 cells and RKO cells were exposed to increasing concentrations of oxaliplatin (0.1–2 μM) over a three-month time-frame, with the final concentration maintained at 2 μM. Human cancer cell lines were cultured at 37 °C in a humidified atmosphere of 5% CO_2_ in MEM, supplemented with 10% fetal bovine serum (FBS), 1% penicillin-streptomycin, L-Glutamine, MEM vitamin solution, sodium pyruvate and MEM non-essential amino acids (Life Technologies, CA). Oxaliplatin resistant cells were maintained in 2 μM oxaliplatin, but were switched to oxaliplatin-free media for at least 24 hours prior to all experimentation. Cells were confirmed to be free of Mycoplasma using the Mycoplasma Detection Test^27^. All experiments were performed at 70% cell confluence with no more than 20 cell passages. Results from all oxaliplatin-resistant cell culture studies were confirmed in at least three independent experiments.

### Drugs and antibodies

Oxaliplatin, N-acetyl-L-cysteine (NAC) and dimethyl sulfoxide (DMSO) were purchased from Sigma-Aldrich, MO. PGE_2_, EP receptor selective antagonists and EP4 receptor agonist were purchased from Cayman Chemicals, MI. Antibodies used for immunoblotting and immunofluorescence were as follows: rabbit anti-mPGES-1 (Abnova, Taiwan), rabbit anti-COX-2, rabbit anti-EP1, rabbit anti-EP2, rabbit anti-EP3, rabbit anti-EP4 (Cayman Chemicals), rabbit anti-cleaved PARP, rabbit anti-phospho-Akt, rabbit anti-Bcl2, rabbit anti-Bax (Cell Signaling Technology, MA), mouse anti-β actin (Sigma). Mouse anti-15-PGDH was a generous gift from Drs. Sanford D. Markowitz and Stephen Fink at Case Western Reserve University.

### Cell viability assay

Cell sensitivity to drugs were assessed using a colorimetric 3-(4,5-Dimethylthiazol-2-yl)-2,5-diphenyltetrazolium bromide (MTT) assay as described previously with minor changes^28^. Briefly, cells were seeded in 48-well plates overnight and treated with or without drugs for 72 hours, followed by MTT staining and colorimetric analysis. IC50 curves were generated with GraphPad Prism (software versions 5.0c and 7.0) using variable slope model.

### Gene knockdown using small interfering RNA (siRNA) and PGE_2_ measurement

Cells were seeded in 6- or 48-well plates overnight. Cell layers were rinsed twice with sterile PBS followed by overnight incubation with OPTI-MEM (Life Technologies) containing K2 transfection reagent (1 μl/100 μl, Biontex, Germany) and siRNA against mPGES-1 (5 nM siGENOME SMARTpool siRNA targeting human PTGES) or control siRNA (5 nM siGENOME Non-Targeting siRNA) (Dharmacon, CO). Media was then changed to MEM supplemented with 10% FBS. After 48–72 h, culture medium was collected and PGE_2_ concentrations were measured by commercial ELISA kit (Cayman) according to manufacturer’s protocol. Cells were harvested for RNA isolation or protein extraction.

### RNA isolation and reverse transcription polymerase chain reaction (RT-PCR)

Total RNA was isolated using the RNeasy Mini kit (Qiagen Inc., Germany). cDNA was synthesized using Superscript III reverse transcriptase (Invitrogen, CA). mRNA expression levels of genes of interest were examined with iTaq Universal SYBR Green Supermix (Bio-Rad Laboratories, Inc., CA) on the ABI-7500 platform (Applied Biosystems, CA). mRNA expression levels of γ-glutamyl-transpeptidase (*GGT*), Glutathione peroxidase (*GPX*), microsomal prostaglandin E synthase-1 (*mPGES-1*), 15-hydroxyprostaglandin dehydrogenase (15-PGDH) and cyclooxygenase-2 (*COX-2*) were normalized to glyceraldehyde 3-phosphate dehydrogenase (*GAPDH*).

### Immunoblotting

Cultured cells were lysed in 1xRIPA buffer supplemented with protease and phosphatase inhibitor (Sigma). The cell lysates were ultra-sonicated and centrifuged. The protein concentration was determined using Protein Assay solution (Bio-Rad). 30-μg of protein was loaded for gel electrophoresis (Bio-Rad) and transferred to Immobilon-P membrane (EMD Millipore, MA). The membranes were blocked in 5% non-fat dry milk in TBST (1xTBS, 0.1% Tween 20) for 1 hour. Blots were incubated with primary antibodies overnight at 4 °C and HRP-conjugated secondary antibodies for 1 hour at room temperature. HRP was visualized with enhanced luminal reagent (EMD Millipore) on ChemiDoc MP imager (Bio-Rad). Images were processed using Image Lab software (Bio-Rad).

### Cell cycle analysis

Drug-treated or control cells were collected and fixed in cold methanol, and stained with 7-aminoactinomycin D (7-AAD) or propidium iodide (PI) (Sigma). After staining, cells were collected and analyzed for DNA content using LSR-II Flow Cytometer (BD Biosciences, CA). All Analyses were performed in triplicate and 50,000 gated events/sample were counted using FlowJo 10.3 software (FlowJo LLC, OR). Cell-cycle stages and apoptosis rates were analyzed using ModFit LT 3.3.11 software (Verity Software House Inc, ME).

### Sphere formation assay

Tumor sphere formation was evaluated as previously described with minor modifications^29^. Briefly, 100 cells were cultured in a 96-well ultra-low attachment surface plate with 100 μL serum-free DMEM/F12 medium containing B27 supplement, 20 ng/mL EGF and 20 ng/mL FGF (Invitrogen) for 7 days. The formation of spheres was evaluated at days 1, 3 and 5 post-seeding by light microscopy and the number of spheres per well were counted at day 7.

### ROS detection

Cellular ROS levels were detected with H_2_DCFDA (Life Technologies) staining as previously described with minor changes^30^. Briefly, drug-treated or control cells were washed twice with serum-free media and then incubated with 2 μM H_2_DCFDA in 2% FBS media at 37 °C for 30 minutes. Following incubation, cells were washed twice with serum-free media, trypsinized and collected for subsequent flow cytometry analysis.

### Glutathione (GSH) assay

Drug-treated or control cells were collected and cell numbers were counted. Cells were ultra-sonicated and centrifuged. Supernatants were de-proteinated and total GSH levels were measured using a commercial glutathione assay kit (Cayman Chemical) according to the manufacturer’s protocol.

### Statistical analysis

Data was analyzed using the Student’s t-test or a one-way analysis of variance (ANOVA) when appropriate using GraphPad Prism (software versions 5.0c and 7). For the MTT assay, 50% inhibitory concentrations of oxaliplatin were calculated and compared using the Extra sum-of-squares F-test. Results were considered as statistically significant at a P-value of less than 0.05. All statistical tested were two-sided.

## Results

### Dysregulation of PGE_2_ metabolism in oxaliplatin -resistant colon cancer cells

**Figure 1:**
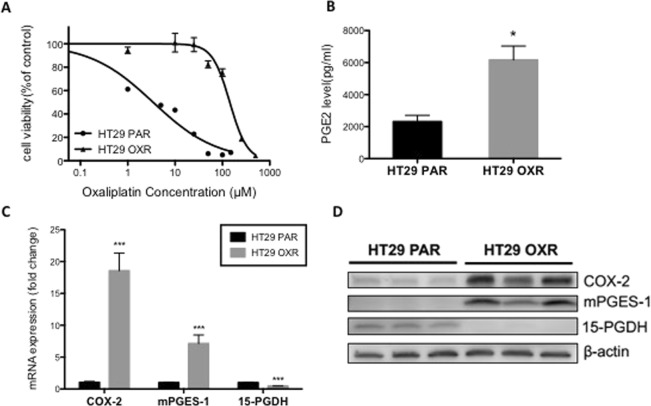
PGE_2_ metabolism is dysregulated in oxaliplatin-resistant cells. (**A**) HT29 cells were treated with varying concentrations of oxaliplatin for 72 hours and cell viability was determined by the MTT assay. Data represent the means +/− SEM of two independent experiments performed in duplicate. The dose-response curves and IC50 values were determined by a non-linear regression fit. ***P < 0.0001 compared with PAR-HT29 cells (extra sum-of-squares F-test). (**B**–**D**) HT29 PAR and OXR cells were maintained in cell culture conditions for 48 hours and then evaluated for basal PGE_2_ metabolism. (**B**) Total secreted PGE_2_ levels in cell culture media were measured using ELISA. (**C**) RT-PCR and (**D**) Western blot analyses for COX-2, mPGES-1 and 15-PGDH mRNA or protein prepared from untreated OXR and PAR cells. Full-length blots are presented in Supplementary Fig. 4. Columns represent the means +/− SEM of quadruplicate samples. *P < 0.05 compared with HT29 PAR cells (Student’s *t*-test).

The human HT29 OXR cells used in these experiments were found to be resistant to oxaliplatin treatment (72 hours), with an IC50 of 134.1 μM *vs*. 3.4 μM in parental (HT29 PAR) cells (P < 0.0001) (Fig. 1A). To determine whether PGE_2_ metabolism is altered in the oxaliplatin-resistant cells, we measured the levels of PGE_2_ in the media from the parental PAR cells and OXR cells. As shown in Fig. 1B, OXR cells produced higher mean levels of PGE_2_ (~3-fold; P < 0.05) compared to the PAR. To explore the basis for the increased concentrations of PGE_2_, we measured the levels of several key metabolic enzymes involved in PGE_2_ production and turnover. Compared to the PAR cells, OXR cells exhibited increased expression of both COX-2 (18-fold; P < 0.001) and the terminal PGE_2_ synthase, microsomal prostaglandin E synthase-1 (mPGES-1) (7-fold increase; P < 0.001) (Fig. 1C,D). Meanwhile, there was a significant reduction (~55%; P < 0.001) in both the mRNA expression and protein levels of 15-PGDH (Fig. 1C,D), the major PGE_2_ catabolic enzyme^31,32^. The increase of COX-2 and mPGES-1 expression levels were not observed in oxaliplatin-resistant cell line RKO OXR in comparison to the parental (RKO PAR) cells, despite significant resistance to oxaliplatin treatment (72 hours), with an IC50 of 13.04 μM *vs*. 5.821 μM in RKO PAR cells (P < 0.0001) (Supplementary Fig. 1A).

### *PTGES* knockdown sensitizes OXR cells to oxaliplatin

To gain further insight into the role of PGE_2_ on oxaliplatin resistance, PGE_2_ synthesis was reduced in OXR cells by siRNA-mediated knockdown of *PTGES*, the gene encoding mPGES-1^33^. As shown in Supplementary Fig. 2A,B, siRNA treatment for 48 hours effectively reduced both mRNA expression and protein levels of mPGES-1, with a concomitant reduction in PGE_2_ synthesis of ~85% (P < 0.001) (Supplementary Fig. 2C). After confirming the efficiency of the siRNA knockdown, we determined the impact of PGE_2_ on cell viability. After a 48-hour siRNA treatment, cells were washed, treated with oxaliplatin for 72 hours and evaluated for viability. As shown in Supplementary Fig. 2D, inhibition of PGE_2_ synthesis increased cell sensitivity to oxaliplatin by ~33%, significantly lowering the IC50 from 55.6 to 37.4 uM (p = 0.0015).

### A selective EP4 receptor antagonist sensitizes resistant cells to oxaliplatin

**Figure 2:**
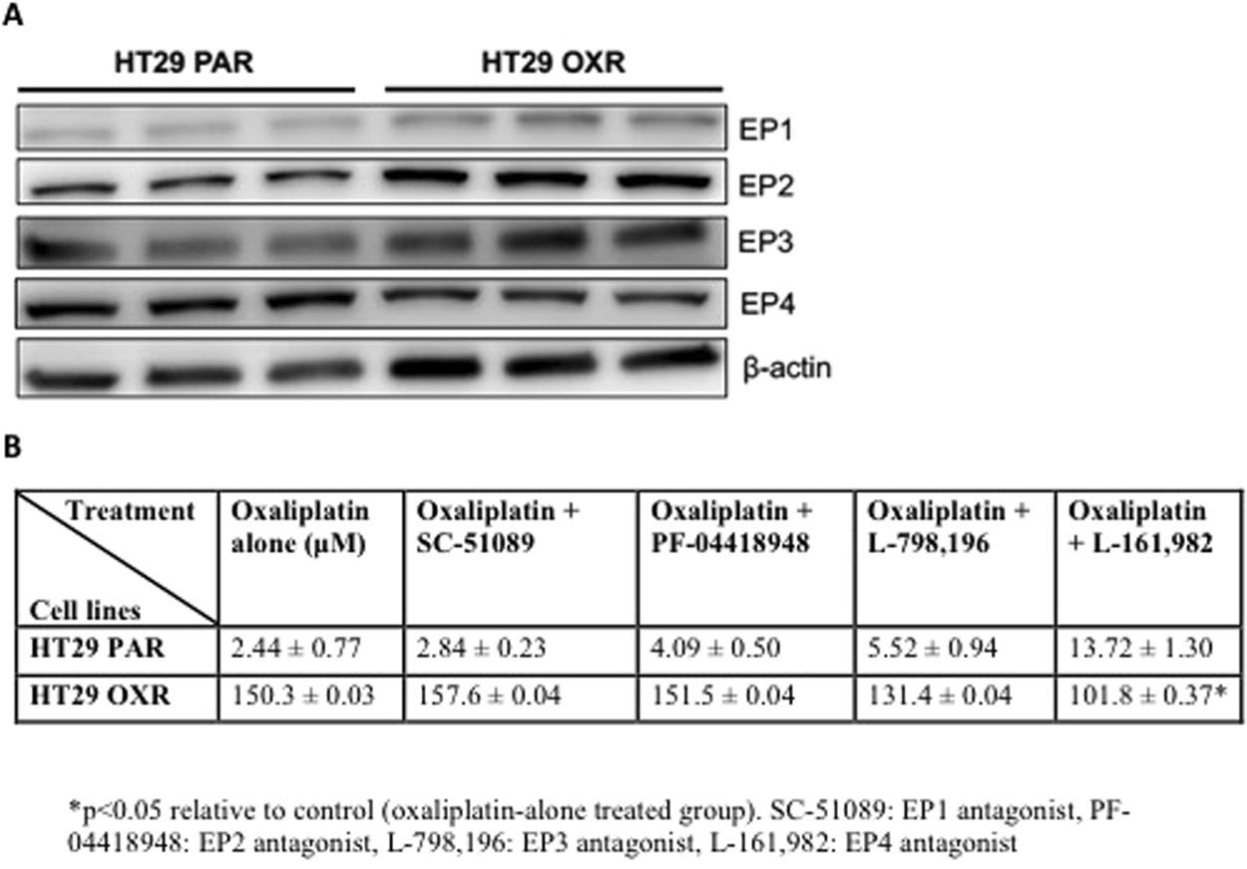
EP receptor status and the effects of EP receptor blockade on cell viability in HT29 OXR and PAR cells. HT29 OXR and PAR cells were tested for their EP receptor expression status and effects of pharmacologic inhibition on oxaliplatin sensitivity. (**A**) Western blot analysis for EP (1–4) receptor expression in untreated PAR and OXR cells. Full-length blots are presented in Supplementary Fig. 5. (**B**) HT29 OXR or PAR cells were treated with increasing concentrations of either oxaliplatin alone (control), or co-treated with 10 μM SC-51089 (EP1-selective antagonist), 10 μM PF-04418948 (EP2-selective antagonist), 10 μM L-798,106 (EP3-selective antagonist) or 10 μM L-161,982 (EP4 inhibitor) for 72 h. Cell viability was assessed using the MTT assay. Data represent the means +/− SEM of two independent experiments performed in duplicate. IC50 values were determined by nonlinear regression fit followed by the extra sum-of-squares F-test. *P < 0.05 compared with the control group (extra sum-of-squares F-test).

The activity of PGE_2_ is largely mediated by its binding to a panel of G-protein-coupled receptors (GPCRs), namely the EP1-4 receptors^15^. We examined the expression levels of the individual EP receptors in PAR and OXR-HT29 cells. As shown in Fig. 2A and Supplementary Fig. 5C, we found comparable levels of the EP receptors in OXR cells and parental controls. Quantification results show a 1.9-fold non-significant (p= 0.46) increase in EP1 and a 1.3-fold non-significant (p = 0.10) increase in EP2 receptor expression in HT29 OXR cells compared to HT29 PAR cells (Supplementary Fig. 5). To determine the effects of individual EP receptor blockade on sensitivity of cells to oxaliplatin, we exposed OXR and PAR cells to a panel of selective EP receptor antagonists (EP1: SC-51089, EP2: PF-04418948, EP3: L-798,106, EP4: L-161,982; 10 μM) in combination with oxaliplatin (10/25/50/100 μM) for 72 hours (Fig. 2B) and found that the EP4 inhibitor, L-161,982, had the most dramatic effect (~1.5-fold decrease; P < 0.05) on cell viability in OXR cells, with no effect in the PAR cells (Fig. 2B). In addition, EP4 receptor blockade by L-161,982 did not affect oxaliplatin resistance in mPGES-1 negative RKO OXR cells, suggesting that the synergistic effect of L-161,982 is dependent upon PGE_2_/EP4 signaling (Supplementary Fig. 1A).

**Figure 3:**
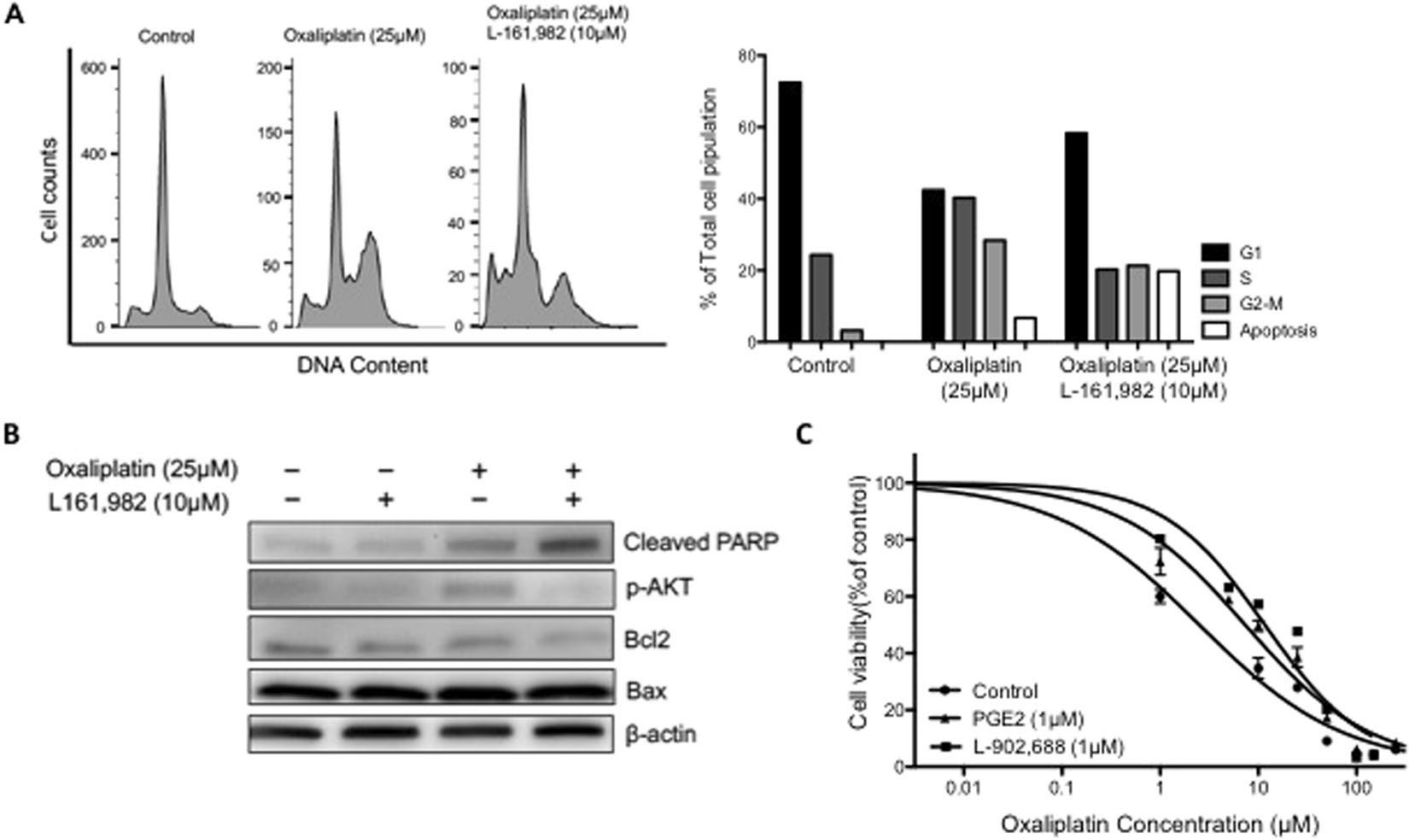
Pharmacologic blockade of the EP4 receptor synergistically enhances oxaliplatin efficacy and increases apoptosis in OXR cells. The effect of EP4 inhibition on oxaliplatin-induced cytotoxicity was tested on HT29 OXR and PAR cells. (**A**) OXR cells were treated with either vehicle control, 25 μM oxaliplatin alone or 25 μM oxaliplatin + 10 μM L-161,982 for 48 hours. Cells were then collected for cell-cycle analysis using flow cytometry. The percentage of cells at each stage and the rate of apoptosis in each group were analyzed with ModFit LT and plotted. (**B**) OXR cells were treated with the indicated drug treatments for 48 hours and cleaved PARP, pAKT, Bcl2 and Bax levels were determined by Western blot analysis. Full-length blots are presented in Supplementary Fig. 6. (**C**) PAR cells were treated with increasing concentrations of oxaliplatin alone (control) or co-treated with 1 μM PGE_2_ or 1 μM L-902,688 (EP4 selective agonist) for 72 hours. Cell viability was assessed using the MTT assay. Data represent the means +/− SEM of two independent experiments performed in duplicate. Dose-response curves and IC50 values were determined by a non-linear regression fit. ***P < 0.0001 compared with control group (extra sum-of-squares F-test).

Oxaliplatin exerts its cytotoxicity by inducing widespread DNA damage^10^, which in turn causes cell cycle arrest and apoptosis^9^. We therefore determined whether EP4 receptor blockade might enhance oxaliplatin-induced cell cycle arrest and cell death. The combination treatment of L-161,982 (10 μM) and oxaliplatin (25 μM) for 48 hours significantly increased the sub-G1 cell population of OXR cells compared to oxaliplatin treatment alone (Fig. 3A). The percentage of cells at each cell cycle stage and the apoptosis rate were then calculated by computer modeling using ModFit LT (Fig. 3A,B). Immunoblotting demonstrated that the levels of PARP cleavage induced by oxaliplatin was also increased by L-161,982, consistent with increased apoptosis of OXR cells (Fig. 3B). In contrast, AKT phosphorylation and the Bcl2/Bax ratio were reduced following the combination treatment (Fig. 3C). Taken together, these results show that selective blockade of the EP4 receptor by L-161,982 enhances oxaliplatin-induced cytotoxicity in OXR cells.

### Pharmacologic activation of EP4 receptor increases viability of human colorectal cancer cells

To determine the generalizability of EP4 receptor activity towards oxaliplatin-induced colorectal cancer resistance, we exposed three additional human CRC cell lines, SW480, Caco-2 and HCT116, to varying concentrations of oxaliplatin and the EP4 receptor agonist (L-902,688). Co-treatment with oxaliplatin and L-902,688 resulted in a significant (P < 0.001) increase in the viability of all three cell lines. The IC50 value increased from 1.86 to 3.59 μM, 7.63 to 26.4 μM, 1.84 to 3.94 μM in SW480, Caco-2 and HCT116 cell lines respectively (Supplementary Fig. 1B).

Finally, we tested the effects of the EP4 receptor agonist on oxaliplatin-induced cytotoxicity in the HT29 parental cells. Specifically, we compared the toxicity of oxaliplatin, oxaliplatin + PGE_2_, and oxaliplatin + L-902,688 on PAR cells. As shown in Fig. 3C, co-treatment of PAR cells with increasing concentrations of oxaliplatin (1 to 100 μM) in the presence of either PGE_2_ or the EP4 receptor agonist, L-902,688 (1 μM), resulted in a significant increase in cell survival, from an IC50 value of 2.5 μM in controls up to 7.3 and 11.2 in PGE_2_ and L-902,688-treated cells, respectively (P < 0.001). These data provide further evidence that activation of EP4 receptor signaling protects against oxaliplatin-induced colon cancer cell death.

### Effects of PGE_2_-EP4 receptor signaling on tumor sphere forming capacity of oxaliplatin resistant cells

**Figure 4:**
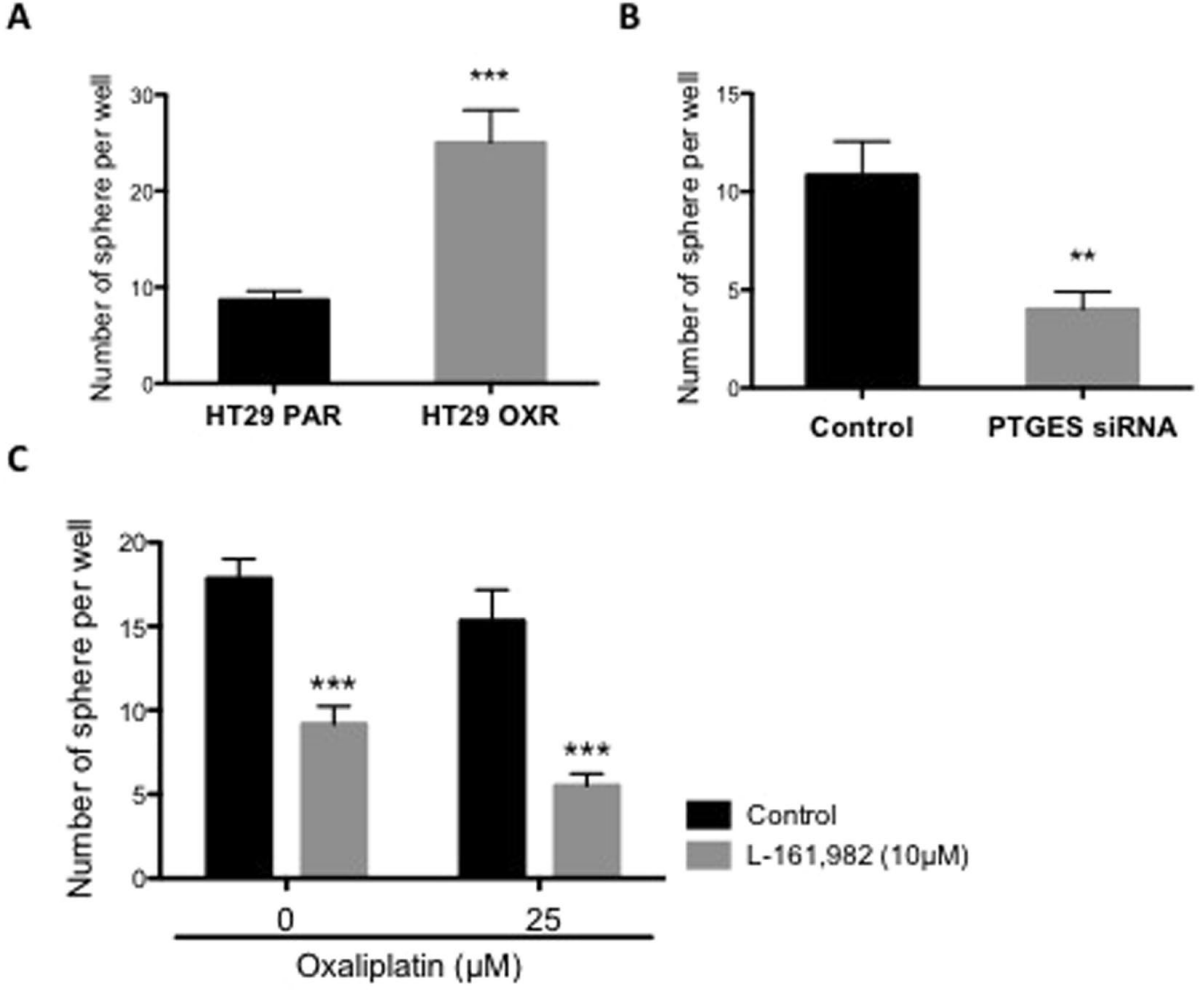
Selective blockade of EP4 significantly reduces tumor sphere formation of HT29 OXR cells. The effect of EP4 inhibition on tumorigenicity was tested in HT29 OXR cells. (**A**) Untreated PAR and OXR cells were plated in an ultra-low attachment 96-well plate (100 cells per well) for 1 week and the numbers of viable tumor spheres per field were counted. (**B**) HT29 OXR cells were treated with *PTGES* siRNA or non-targeting (NT) siRNA for 48 hours, followed by a tumor sphere formation assay (Materials and Methods). Columns, means of samples of two independent experiments; bars, SEM. **P < 0.01, ***P < 0.001 (Student’s *t*-test). (**C**) OXR cells were seeded in an ultra-low attachment plate and treated with the indicated combination of oxaliplatin and the EP-4 inhibitor, L-161,982 for 1 week. At day 7, the number of viable tumor spheres per field were counted. Columns, means of samples of two independent experiments; bars, SEM. ***P < 0.001 (One-way ANOVA).

An important mechanism for chemoresistance is the enrichment of the cancer stem cell (CSCs) population within the tumor mass^22,34^. Earlier studies of oxaliplatin resistance identified an increase in the number of CSCs in oxaliplatin-resistant CRC cell lines^6,26^. Consistent with these earlier findings, we found that OXR cells showed elevated tumor spheroid-forming capacity, a useful surrogate for modeling CSC response to anti-cancer agents (Fig. 4A). To further examine the potential effects of PGE_2_ on tumor sphere growth, we tested the ability of OXR cells to form tumor spheres in *PTGES* knockdown cells (Fig. 4B). Indeed, the number of tumor spheres was significantly reduced (~2-fold; P < 0.01) in the *PTGES* knockdown cells. Finally, we tested the EP4 inhibitor, L-161,982 and found reduced tumor spheroid formation, both in the presence and absence in oxaliplatin (Fig. 4C). These findings demonstrate the role of PGE_2_ and EP4 in maintaining the CSC population and contributing to oxaliplatin resistance.

### Effects of PGE_2_-EP4 receptor signaling on oxidative stress in oxaliplatin -resistant cells

**Figure 5:**
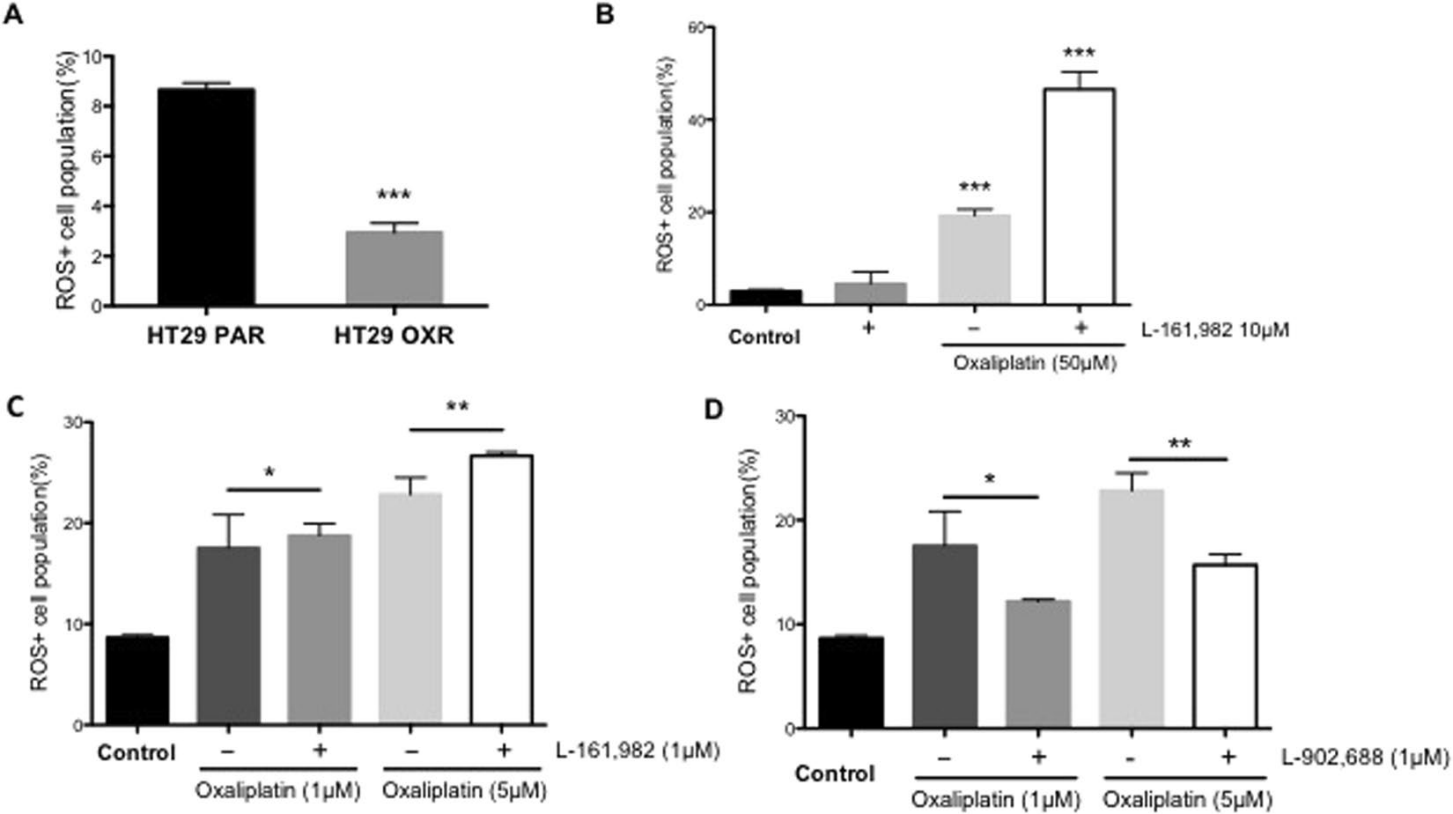
The synergistic effect of L-161,982 on oxaliplatin efficacy in HT29 OXR cells is mediated by intracellular reactive oxygen species accumulation. HT29 PAR and OXR cells were examined for baseline ROS formation and the effects of EP4 receptor blockade on oxaliplatin-induced ROS. (**A**) Intracellular ROS levels were measured in untreated PAR and OXR cells using the H_2_DCFDA fluorescence assay. Columns, means of triplicate samples; bars, SEM. ***P < 0.001 (Student’s *t*-test). (**B**) OXR cells were treated with either L-161,982 (10 μM) or oxaliplatin (50 μM), singly, or in combination for 48 hours and ROS formation was measured. Columns, means of triplicate samples; bars, SEM. ***P < 0.0001 (One-way ANOVA). (**C**) HT29 PAR cells were treated with the indicated concentrations of oxaliplatin (1 μM or 5 μM), individually, or in combination with L-161,982 (10 μM) for 48 hours and ROS formation was measured. Data represent the means +/− SEM of triplicate samples (**P < 0.01, n.s., not significant; data were analyzed by a one-way analysis of variance). (**D**) To determine whether EP4 receptor activation may affect cell survival, HT29 PAR cells were treated with the indicated concentrations of oxaliplatin (1 μM or 5 μM), individually, or in combination with the EP4 receptor agonist, L-902,688 (1 μM). Data represent the means +/− SEM of triplicate samples (one-way analysis of variance).

Recent studies indicate that oxidative stress plays an important role in oxaliplatin-induced cytotoxicity^30,35^. In the following experiment, we evaluated the possibility that inhibition of EP4 signaling might enhance oxaliplatin-induced cytotoxicity by increasing the levels of oxidative stress. Intracellular ROS levels were measured in both PAR and OXR cells using the H_2_DCFDA fluorescence assay. As shown in Fig. 5A, compared to HT29 PAR cells, basal ROS levels were significantly lower (~60% of PAR; P < 0.001) in OXR cells under normal culture conditions. Meanwhile, the levels of both GSH (~1.2-fold) and *GGT* (~5-fold) were significantly (P<0.05) higher in the HT29 OXR vs. HT29 PAR cells, respectively (Supplementary Fig. 3), indicating altered REDOX status in the oxaliplatin-resistant cells. We next sought to determine whether oxaliplatin treatment, combined with EP4 receptor blockade, may further elevate ROS levels in the OXR-HT29 cells. As shown in Fig. 5B, OXR cells were treated for 48 hours with either L-161,982 (10 μM) alone, or in combination with oxaliplatin (50 μM). While L-161,982 treatment alone failed to significantly elevate ROS levels, there was a significant increase (~10-fold; P < 0.0001) in ROS when combined with oxaliplatin (Fig. 5B). In contrast, although ROS levels were also increased in PAR HT29 cells by treatment with 1 and 5 μM oxaliplatin, there was no further increase in ROS upon EP4 receptor blockade with L-161,982 (Fig. 5C). Finally, treatment of the parental HT29 cells with the EP4 receptor agonist, L-902,688, significantly attenuated oxaliplatin-induced ROS formation (Fig. 5D).

Finally, the effects on ROS formation of the EP4 receptor agonist, L-902,688, was tested in the PAR cells. As shown in Fig. 5D, the addition of L-902,688 (1 μM) to oxaliplatin treatment of PAR cells (1 and 5 uM) caused a significant suppression of oxaliplatin-induced ROS elevation (Fig. 5D) corresponding to an increase in cell viability as shown earlier (Fig. 3C). This suggests that EP4 signaling promotes oxaliplatin resistance in human colon cancer cells through suppression of oxidative stress.

### Effects of PGE_2_-EP4 receptor signaling on antioxidant enzyme expression

**Figure 6:**
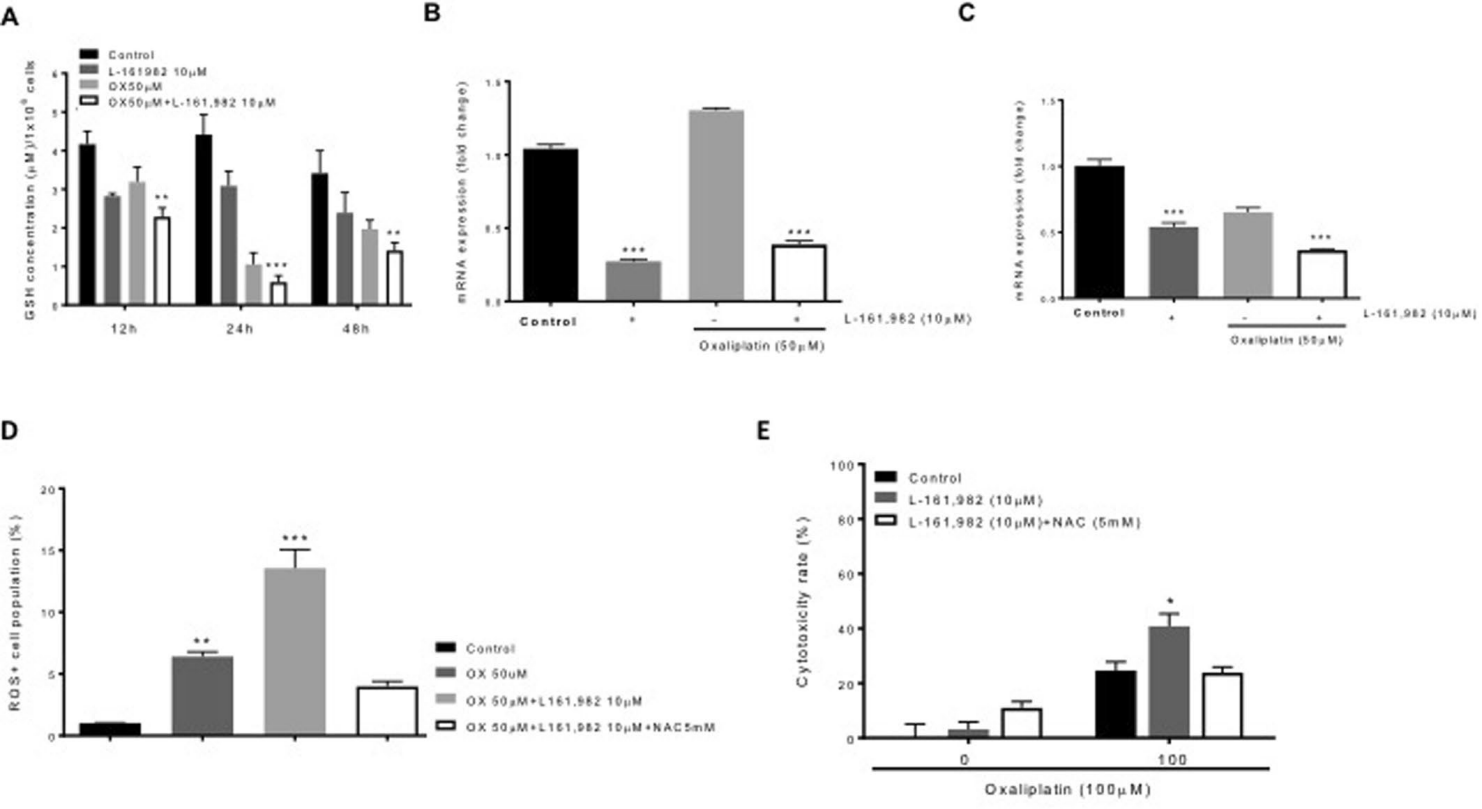
Effects of PGE_**2**_-EP4 receptor signaling on REDOX status. HT29 OXR cells were examined for ROS clearance and the effects of antioxidant enzyme expression on oxaliplatin-induced cytotoxicity. HT29 OXR cells were treated with the indicated combinations of oxaliplatin (50 μM) and the EP4 receptor antagonist, L-161,982 (10 μM) for 48 hours. (**A**) The levels of cellular glutathione (GSH) in HT29 OXR cells were measured at the times indicated. Columns represent the means +/− SEM of two independent experiments; bars, SEM. ***P < 0.0005 (one-way analysis of variance). (**B**,**C**) Total RNA was extracted from OXR-HT29 cells and mRNA expression levels of *GGT1* (**B**) and *GPX2* (**C**) were measured by RT-PCR analysis. Columns represent the means +/− SEM of two independent experiments. ***P < 0.0001 (one-way analysis of variance). (**D**) HT29 OXR cells were treated with the indicated combination of oxaliplatin (50 uM) and L-161,982 (10 uM) and NAC (5 mM) for 48 hours followed with H_2_DCFDA staining. The cellular ROS level was measure by flow cytometry analysis. Columns represent the means +/− SEM of two independent experiments. ***P < 0.0001 (one-way analysis of variance). (**E**) HT29 OXR cells were treated with the indicated combination of oxaliplatin and L-161,982 and NAC for 72 hours, followed by the MTT assay. Cell viability rate was defined as the percentage of living cells in each group compared to untreated cells. Columns represent the means +/− SEM of two independent experiments. *P < 0.05 (one-way analysis of variance, in comparison to both control group and NAC group).

To determine the impact of oxaliplatin and EP4 signaling on antioxidant status in OXR cells, we first monitored GSH levels during a 48-hour treatment interval with oxaliplatin, with or without the addition of the EP4 antagonist, L-161,982. As shown in Fig. 6A, the combined treatment with 50 μM oxaliplatin and 10 μM L-161,982 resulted in a significant reduction in GSH concentrations at 12, 24 and 48 hours (~1.8-, 9.0- and 2.3-fold, respectively; P < 0.05). We next examined the effects of a 48-hour treatment of OXR cells using 10 μM L-161,982, alone and in combination with 50 μM oxaliplatin, on *GGT* and *GPX* mRNA expression. As shown in Figure 6B and C, treatment with 10 μM L-161,982 alone for 48 hours significantly reduced the expression of both *GGT* and *GPX* (~70% reduction; P < 0.0001 and ~50% reduction; P < 0.0001, respectively) compared to non-treated (control) cells. Surprisingly, the combination of 50 μM oxaliplatin with 10 μM L-161,982 did not produce a further decline in *GGT* expression, although the drug combination did, in fact, enhance the reduction in *GPX* levels.

Finally, we tested the effects of the potent anti-oxidant drug, N-acetyl cysteine (NAC), on oxaliplatin-induced ROS formation in OXR cells. As shown in Fig. 6D, 50 μM oxaliplatin caused a significant increase in ROS levels, which were augmented by the addition of L-161,982 (~13-fold; P < 0.05). The addition of 5 mM NAC, however, was able to significantly reverse (~70% reduction; P < 0.0001) the ROS-enhancing effect of oxaliplatin + L-161,982. Finally, we tested the effects of NAC on L-161,982-induced cytotoxicity in OXR cells in the presence and absence of oxaliplatin (Fig. 6E). In the absence of oxaliplatin, NAC had no effect on cell viability. However, NAC significantly reduced cytotoxicity when cells were treated with a combination of L-161,982 and oxaliplatin.

## Discussion

The acquisition of cancer cell resistance is a critical factor limiting the therapeutic efficacy of oxaliplatin for CRC. While resistance to oxaliplatin may be acquired through a number of mechanisms, here we report that the increased production of PGE_2_ may play a significant role in resistance to chemotherapy. We show that the increase in PGE_2_ formation in oxaliplatin-resistant colon cancer cells is a result of elevated expression of COX-2 and mPGES-1, with a concomitant decline in the expression of the PGE_2_ degrading enzyme, 15-PGDH. We further demonstrate that paracrine signaling through the EP4 receptor increases cancer cell resistance by reducing the levels of oxidative stress induced by oxaliplatin. These findings raise the possibility that EP4 receptor may offer a viable therapeutic target for maintaining cancer cell sensitivity to oxaliplatin treatment.

The EP receptor family is comprised of four GPCRs that signal through distinct cellular pathways, mediating a divergent set of biological responses to PGE_2_^36^. Genetic ablation of individual EP receptor subtypes in mouse models have provided insight into the complex and often overlapping role of these receptors in CRC pathogenesis^15^. For example, Sonoshita and colleagues^37^ determined that homozygous deletion of the gene coding EP2 reduces intestinal polyposis in *ApcΔ716* mice, whereas genetic inactivation of the EP1 and EP3 receptors failed to elicit protection. In studies using azoxymethane to induce colon cancer, it was shown that EP1 receptor knockout mice developed fewer colonic lesions than their wild-type counterparts^38^, whereas EP3 knockout mice showed enhanced colon carcinogenesis^39^. Our studies have revealed an important role of EP4 on oxaliplatin resistance. The EP4 receptor has previously been shown to promote colon cell proliferation and survival, tumor metastasis, and suppression of antitumor immunity, both in cell culture systems and in mouse cancer models^40–42^. Our studies establish a functional role for the EP4 receptor that directly contributes to the acquisition of drug resistance. A number of mechanisms have been invoked to explain the development of chemoresistance to oxaliplatin treatment in CRC. One well-established mechanism involves the activation of multi-drug resistance (MDR), which effectively reduces oxaliplatin concentrations in cancer cells by increasing drug efflux through MDR1^43–45^. In the present study, although we found a modest, non-significant increase (~30%, P = 0.38) in MDR1 expression in RKO OXR cells in comparison to the RKO parental cells (Supplementary Fig. 1A), MDR1 levels were unchanged in OXR cells. These findings raise the possibility that alternative mechanisms may contribute to oxaliplatin resistance in HT29 cell lines.

Recently, it has been demonstrated in a number of different malignancies that the enrichment of CSCs within the tumor mass are associated with chemoresistance^26,46,47^. In fact, Dubois and co-workers^48^ have recently shown that PGE_2_/EP4 signaling may induce the expansion and/or survival of CSCs in CRC. Thus in the present study, we explored the possibility that inhibition of PGE_2_ synthesis and/or EP4 receptor signaling might affect the behavior of CSCs derived from OXR cells. Compared to the HT29 parental cells, tumor sphere formation from OXR cells was markedly elevated. Furthermore, siRNA-mediated knockdown of *PTGES* resulted in a significant reduction in the number of tumor spheres formed from OXR cells. In addition, EP4 blockade with L-161,982 also led to a marked reduction in tumor sphere formation as well. Overall, our findings uncover an important role of PGE_2_-EP4 signaling in maintaining cancer stem cell viability, an effect that may provide an underlying mechanism for oxaliplatin resistance.

A key factor that may contribute to CSC enrichment is the acquisition of increased antioxidant capacity, a common feature of cancer cells^49^. To overcome this metabolic adaptation, certain anticancer agents, including the platinum coordination complexes and antimetabolites, induce mitochondrial dysfunction, thereby generating extremely high levels of ROS, which in turn causes significant DNA damage and cell death^50^. Resistant cancer cells, however, develop cellular mechanisms that enable them to maintain lower levels of ROS relative to chemo-naïve cells^49^. This resistance mechanism is clearly invoked in the case of oxaliplatin^35,51^. In the present study, we have shown that HT29 OXR cells maintain a remarkably lower cellular pools of ROS compared to the HT29 parental cells Fig. 5A. This metabolic phenotype is accompanied by a concomitant elevation in cellular GSH levels indicating an acquired altered redox status in OXR cells. Our data further demonstrate that reduced ROS levels in the OXR cells is mediated, in part, through altered PGE_2_/EP4 signaling. In fact, an association between PGE_2_/EP4 signaling and ROS metabolism was recently reported by Mo and colleagues^52^, who found that EP4 receptor blockade by L-161,982 in myoblasts significantly increased cellular ROS formation and inhibited cell proliferation^52^. Whether this metabolic adaptation can be reversed during the course of chemotherapy remains to be evaluated.

In summary, we have demonstrated a critical role of PGE_2_/EP4 receptor signaling in promoting oxaliplatin resistance in human colorectal cancer cells. Mechanisms include the enforced attenuation of cellular ROS, as well as potential cancer stem cell enrichment, evidenced by enhanced tumor sphere tumor formation. While previous studies have demonstrated the pro-tumorigenic effects of PGE_2_/EP4 signaling in CRC progression^42,53^, our work provides the first direct evidence that PGE2-mediated activation of EP4 signaling is directly related to oxaliplatin resistance. The development of the EP4 receptor as a pharmacologic target for enhancing oxaliplatin efficacy provides the potential for a more durable therapeutic response in advanced CRC patients.

## Supplementary information

Inhibition of PGE2/EP4 receptor signaling enhances oxaliplatin efficacy in resistant colon cancer cells through modulation of oxidative stress

## Data Availability

All data generated or analysed during this study are included in this published article (and its Supplementary Information Files).
