# Supplementary material for: Inhibition of PGE2/EP4 receptor signaling enhances oxaliplatin efficacy in resistant colon cancer cells through modulation of oxidative stress
Source: Sci Rep. 2019 Mar 20;9:4954. doi: 10.1038/s41598-019-40848-4 (PMC6427013; doi:10.1038/s41598-019-40848-4)

Inhibition of PGE_2/EP4 receptor signaling enhances oxaliplatin efficacy in resistant colon cancer cells through modulation of oxidative stress

Huakang Huang[1], Oladimeji Aladelokun[1], Takayasu Ideta[1], Charles Giardina[2], Lee M. Ellis[3], and Daniel W. Rosenberg[1]

1. Center for Molecular Oncology, University of Connecticut Health, 263 Farmington Ave, CT
2. Department of Cell and Molecular Biology, University of Connecticut, Storrs, CT
3. Department of Surgical Oncology, University of Texas MD Anderson Cancer Center, Holcombe Boulevard, Houston, Texas

Corresponding Author: Daniel W. Rosenberg, Center for Molecular Oncology, University of Connecticut Health, 263 Farmington Ave, CT, 06030-3101. Ph:860-679-8704, Fax:860-679-7639, Email: rosenberg@uchc.edu

Supplementary Figure 1A. Oxaliplatin resistance in mPGES-1-negative RKO cell lines is independent of PGE_2/EP4 signaling (i) RKO cells were treated with varying concentrations of oxaliplatin for 72 hours and cell viability was determined by the MTT assay. Data represent the means +/- SEM of two independent experiments performed in duplicate. The dose-response curves and IC50 values were determined by a non-linear regression fit. ***P<0.0001 compared with RKO PAR cells (extra sum-of-squares F-test). (ii) RT-PCR analyses for mPGES-1 mRNA prepared from untreated OXR and PAR cells. Columns represent the means +/- SEM of quadruplicate samples. (iii) Western blot analyses for COX-2 and mPGES-1 protein prepared from untreated HT29 OXR, HT29 PAR, RKO OXR and RKO PAR cells. (iv) RKO OXR or PAR cells were treated with increasing concentrations of either oxaliplatin alone (control), or co-treated with 1 μM L-161,982 for 72h. Cell viability was assessed using the MTT assay. Data represent the means +/- SEM of two independent experiments performed in duplicate. Dose-response curves and IC50 values were determined by nonlinear regression fit followed by the extra sum-of-squares F-test. (v) RT-PCR analyses for MDR-1 mRNA prepared from untreated OXR and PAR cells. Columns represent the means +/- SEM of quadruplicate samples; bars, SEM. P=0.2857 (Student's *t*-test).

Supplementary Figure 1B. Pharmacologic activation of EP4 receptor increases viability of colon cancer cell lines. Human colon cancer cell lines were treated with varying concentrations of oxaliplatin for 72 hours and cell viability was determined by the MTT assay. Data represent the means +/- SEM of experiments performed in duplicate. The dose-response curves and IC50 values were determined by a non-linear regression fit. ***P<0.0001 compared with control (oxaliplatin-alone treated) cells (extra sum-of-squares F-test). (i) SW480 (ii) Caco-2 (iii) HCT116 cancer cell lines

Supplementary Figure 2. siRNA silencing of *PTGES* reduces PGE_2 levels and lowers oxaliplatin resistance in HT29 OXR cells. HT29 OXR cells were treated with siRNA for 48 hours. (A) RT-PCR and (B) Western blot analyses of mPGES-1 expression in OXR cells after a 48-hour treatment with *PTGES* siRNA or non-targeting (NT) siRNA (0.1μg siRNA per $2.5x10^4$ cells). (C) PGE_2 levels were measured in the media after a 48-hour treatment with *PTGES* siRNA or non-targeting (NT) siRNA. Columns, means of quadruplicate samples; bars, SEM. **P<0.01, ***P<0.001 compared with NT siRNA-treated cells (Student's t-test). (D) OXR cells were treated with increasing concentrations of oxaliplatin for 72 hours after *PTGES* siRNA or non-targeting (NT) siRNA treatment (0.1μg siRNA per $2.5x10^4$ cells). Cell viability was assessed using the MTT assay. Cytotoxicity was defined as the percentage of dead cells in oxaliplatin-treated cells compared to untreated cells. Each data point represents the mean value of triplicate samples +/- SEM. IC50 value was determined by a nonlinear regression fit. **P<0.005 compared with NT siRNA treated group (extra sum-of-squares F-test).

Supplementary Figure 3. REDOX status is altered in HT29 OXR cells. GSH levels and antioxidant gene expression were measured in HT29 PAR and OXR cells as described under Materials and Methods. (A) Cells were maintained in standard culture conditions for 48 hours and then evaluated for cellular levels of GSH. Columns represent the mean +/- SEM of two independent experiments; bars, SEM. *P<0.05 (Student's *t*-test). (B) Total RNA was extracted from HT29 PAR and OXR cells and mRNA expression levels of GGT1 and GPX2 were

measured by RT-PCR analysis. Columns, mean of triplicate samples; bars, SEM. ***P<0.0001 (Student's *t*-test).

Supplementary Figure 4. Western blot analyses for COX-2, mPGES-1 and 15-PGDH. As described under Materials and Methods, Western blot analysis was performed using protein samples prepared from untreated OXR and PAR cells.

Supplementary Figure 5. Western blot analysis for EP receptor expression. (A) EP1-4 expression was determined in untreated PAR and OXR cells. (B) Quantification analysis of the Western blots shows that there is no significant difference in the levels of EP receptors 1-3 in HT29 OXR cells compared to HT29 PAR cells. However, HT29 OXR cells have a significant reduction (1.7-fold; P<0.05) in the levels of the EP4 receptor compared to the HT29 parental cell line.

Supplementary Figure 6. Western blot analysis for apoptosis markers. OXR cells were treated with the indicated drug treatments for 48 hours. Cleaved PARP and pAKT levels were determined by Western blot analysis. Original image was taken (A) under an exposure time of 30 sec and (B) under an exposure time of 75 sec. In addition, Bcl2 and Bax levels were determined by Western blot analysis. Original image taken (C) under an exposure time of 50 sec, and (D) under an exposure time of 200 sec.

Supplementary Figure 1A

i

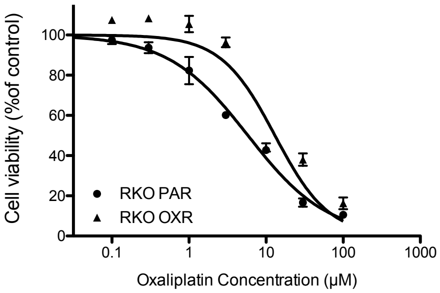

ii

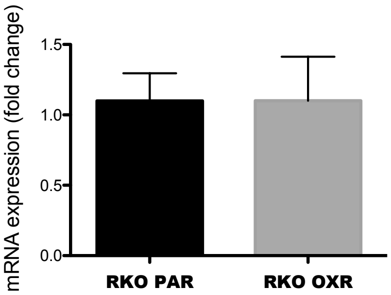

iii

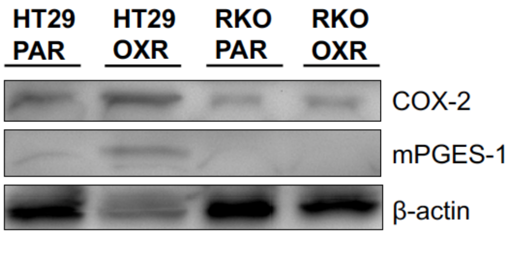

iv

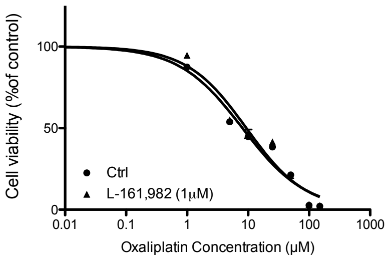

v

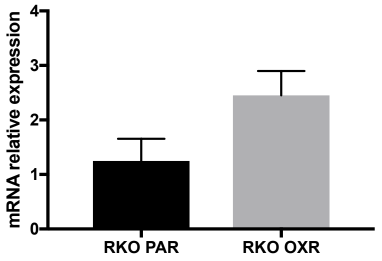

Supplementary Figure 1B

i

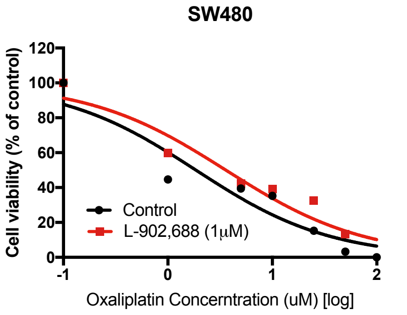

ii

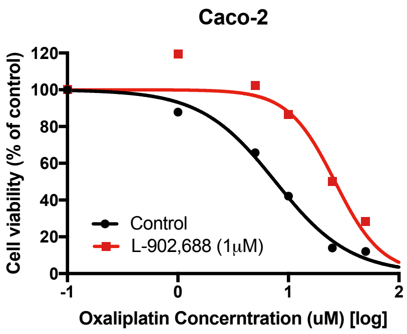

iii

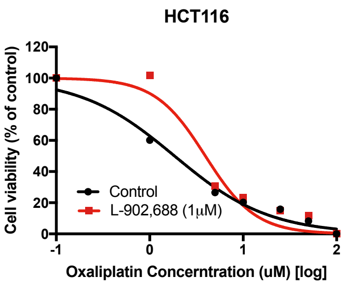

Supplementary Figure 2.

A

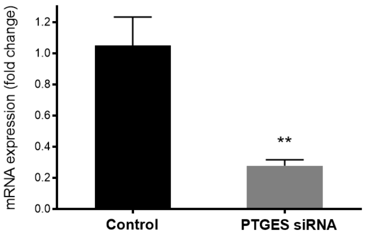

B

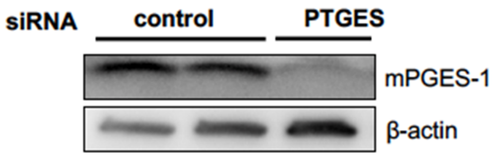

C

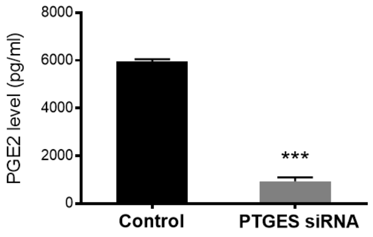

D

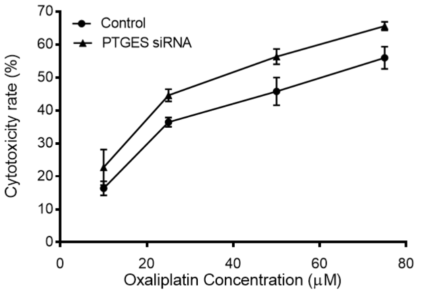

Supplementary Figure 3

A

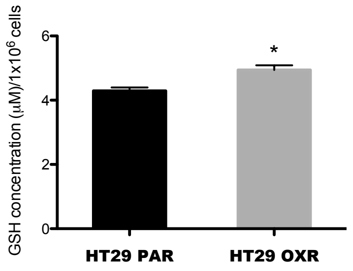

B

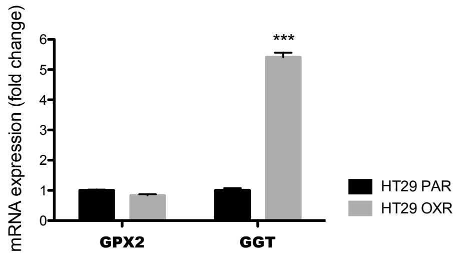

Supplementary Figure 4

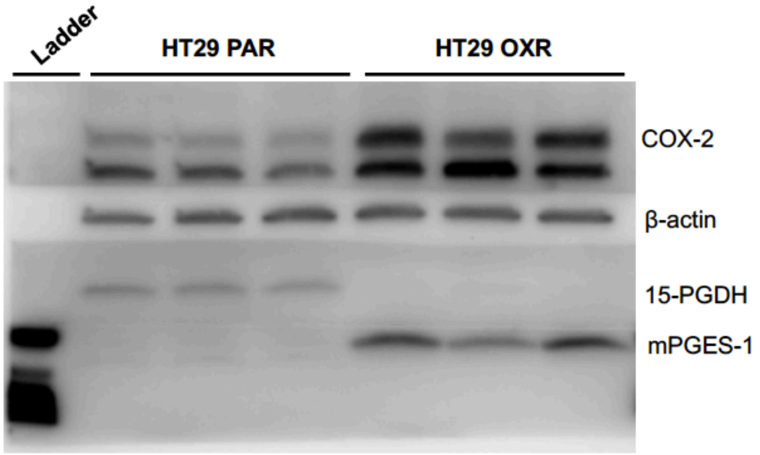

Supplementary Figure 5

A

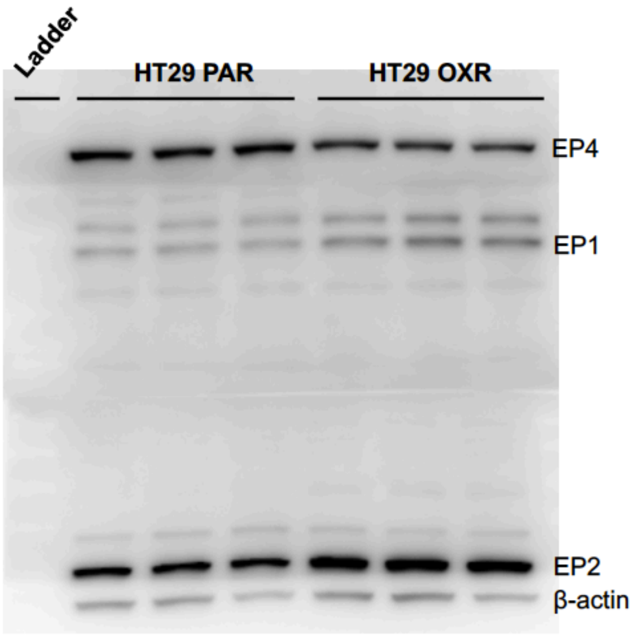

B

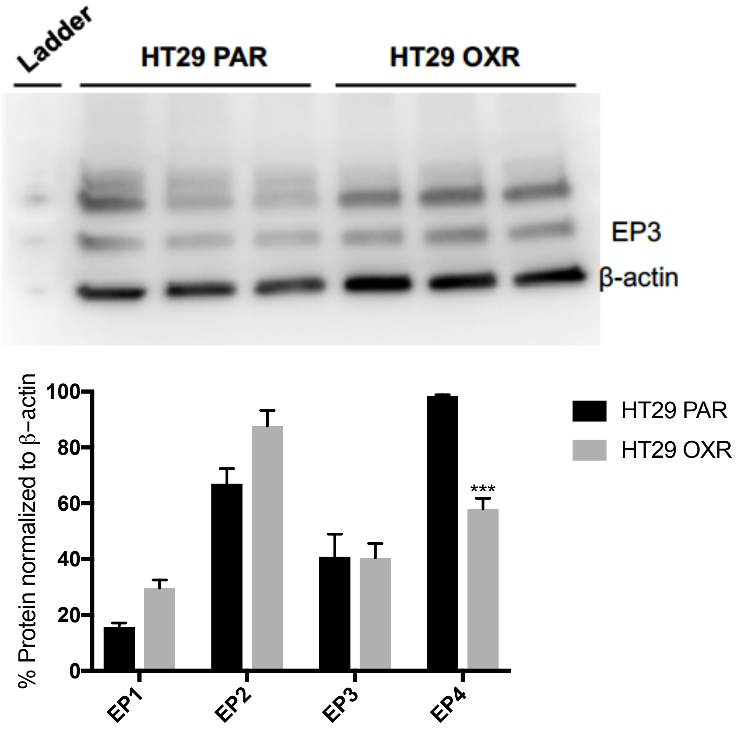

Supplementary Figure 6

A

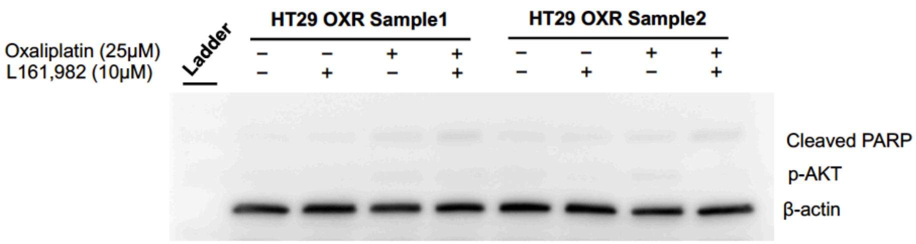

B

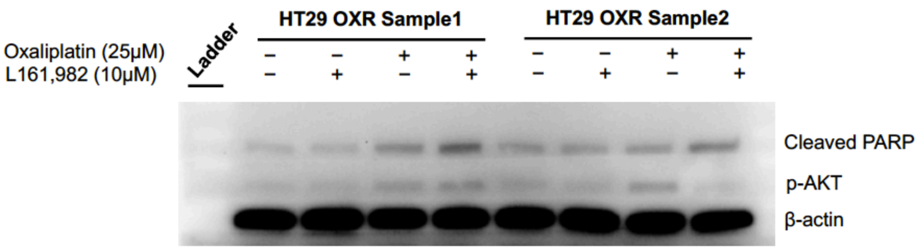

C

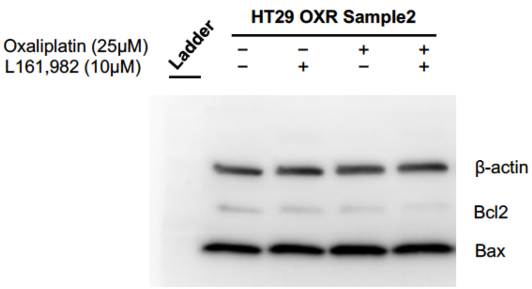

D

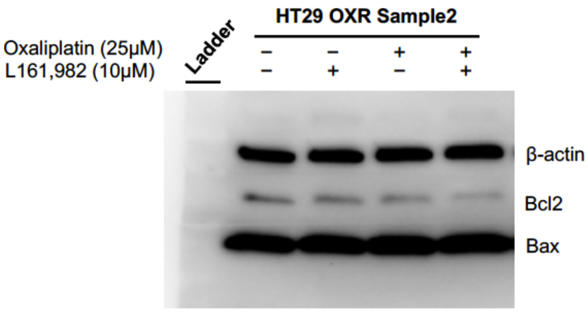

Supplement: Supplementary file 1 — Inhibition of PGE2/EP4 receptor signaling enhances oxaliplatin efficacy in resistant colon cancer cells through modulation of oxidative stress [file 41598_2019_40848_MOESM1_ESM.pdf]
